# Menthol in Acute Rhinitis, Etc.

**Published:** 1890-03

**Authors:** 


### Menthol in Acute Rhinitis, and Other Affections of the Throat and Nose.

According to the experience of Dr. Lennox Browne, of London, in the *Medical Press and Circular*, Jan. 8, 1890, the vapor of menthol checks, in a manner that is hardly less than marvelous, acute colds in the head, and is also to be recommended with a certainty of success, if used on its first onset, in arresting or as a preventive of infection in epidemic influenza, and this even for cases in which the nasal symptoms commonly associated with the word influenza are not manifested.

Menthol exerts its action in the following manner:

1. It stimulates to contraction the capillary blood-vessels of the passages of the nose and throat, always dilated in the early stages of head cold and of influenza.

2. It arrests sneezing and rhinal flow.

3. It relieves, and indeed dissipates, pain and fullness of the head by its analgesic properties, so well known by its action when applied externally to the brow in cases of *tic douloureux*.

4. It is powerfully germicide and antiseptic. It thus kills the microbe of infection, and prevents dissemination.

The remedy may be employed by means of a general impregnation of its vapor through a room or house, or locally to the nostrils and air passages; for both which purposes there are several methods.

1. A 10 to 20 per cent. solution of menthol in almond oil, in liquid vaesline, or in one of the many other odorless paraffin compounds, can be sprayed into the nose or throat, or about a room.

2. By placing twenty or thirty grains in an apparatus specially designed by Rosenberg for administering the drug in cases of laryngeal consumption by inhalation, in the form of vapor mingled with steam.

3. By placing a similar amount or one or two drachms of the oily solution in a Lee's steam draft inhaler, or bronchitis kettle.

4. By placing a saucer of water containing a similar quantity of the crystals over a gas burner in the hall, by means of which the whole house is kept constantly permeated with the drug.

5. But by far the most convenient method for personal use is to carry always the ingenious pocket menthol inhaler known as Cushman's, which should be used not only on the first approach of an attack, but three or four times a day during an epidemic, and always in cold-catching weather by those subject to head colds.

The instrument consists of a glass cylinder four inches in length, half an inch in diameter, and open at both ends. The tube contains crystals of menthol closely packed and prevented from escape by perforated zinc and cork. The opening at one end is twice the size of the other, the larger being intended for inhalatlon by the mouth, the smaller for the nostril. It is not to be simply smelt, but well sniffed or inhaled, so as to cause some tingling or smarting, a sensation which is quickly followed by that of coolness and openness of the previously "stuffed" and heated nostril.

